# Editorial: Controversies in growth hormone treatment and diagnosis

**DOI:** 10.3389/fendo.2022.1013872

**Published:** 2022-09-13

**Authors:** Robert Rapaport, Martin O. Savage

**Affiliations:** ^1^ Kravis Children’s Hospital and Icahn School of Medicine at Mount Sinai, NY, United States; ^2^ William Harvey Research Institute, Queen Mary, University of London, London, United Kingdom

**Keywords:** growth, growth hormone, GH deficiency, short stature, GH treatment

The diagnosis and treatment of pediatric growth disorders encompasses a wide range of topics including diagnostic criteria, including genetic analysis, indications for therapy with recombinant human growth hormone (rhGH) and efficacy and optimal benefit from this therapy. This Research Topic issue includes 19 articles embracing all of these components and gives a rich account of the current state-of-the-art of growth disorder management. In terms of diagnosis, assessment of GH secretion is a controversial field due to the relative inaccuracy of GH stimulation tests. The value of GH testing is addressed by Yau and Rapaport and by Ibba and Loche who cites evidence of GH deficiency without the need to perform formal stimulation tests. An appraisal of the electronic computer-aided bone age diagnosis system, a key factor in short stature evaluation, is discussed and a high degree of confidence reported in this new technology. The genetic components of growth disorders is widely described by several authors, notably with descriptions of genetic syndromes such as brachydactyly, collagen gene mutations, NPR2 gene variants, GH resistance and ring chromosome 15 syndrome. The response of several of these disorders, including children with idiopathic short stature, to treatment with rhGH is reported. The well known but rarely documented or discussed gender and racial disparities in the evaluation and treatment of short stature and GH Deficiency is addressed in a brief review.

Therapy with rhGH is approved by the FDA and European Medicines Agency (EMA) in GH deficient children and several non-GH deficient disorders such as Turner syndrome and short stature related to birth size small for gestational age. The optimization of this therapy has challenged clinicians since its introduction in 1985. The enhancement of height gain using a combination of rhGH and GnRH analogues to suppress skeletal maturation is elegantly debated by Wit. Two further components of rhGH therapy are safety and adherence to the treatment regimen. Safety is discussed in two articles with reassuring conclusions, one related to all-cause mortality and cancer-risk and the second a broad overview of safety and discussion of the need for long-term clinical surveillance by Cianfarani. The second component of adherence to rhGH therapy is addressed in a systematic literature review of the data on injectable treatment in a range of chronic conditions and an objective account of patients’ perception of the use of the electronic autoinjector Easypod™ which is reported to be associated with high rates of adherence.

The final two articles relate first to the important but rare disorder of severe primary IGF-1 deficiency, or GH resistance, which is approved for treatment with rhIGF-1. The topic discussed is the effect of rhIGF-1 therapy on pubertal timing and growth dynamics with data generated from the European Increlex^®^ Growth Forum Database Registry. Finally, a comprehensive appraisal of current opinions on the effect of long-acting rhGH therapy, which is about to enter clinical paediatric practice, is discussed by Miller.

The Research Topic issue presents balanced, objective and nonpromotional discussions of current controversial topics of clinical relevance. Emphasis is given to developments in genetic diagnosis of rare syndromes, which nevertheless present clinical challenges, and to topical issues such equity in diagnosis and treatment as well as the impact of long-acting rhGH. We are confident that these articles will be of value to clinicians responsible for management of growth disorders and therefore positively impact patient care.

## Author contributions

MS drafted initial outline. RR edited and finalized manuscript. All authors contributed to the article and approved the submitted version.

## Conflict of interest

The authors declare that the research was conducted in the absence of any commercial or financial relationships that could be construed as a potential conflict of interest.

## Publisher’s note

All claims expressed in this article are solely those of the authors and do not necessarily represent those of their affiliated organizations, or those of the publisher, the editors and the reviewers. Any product that may be evaluated in this article, or claim that may be made by its manufacturer, is not guaranteed or endorsed by the publisher.

